# Perceptions and Earliest Experiences of Medical Students and Faculty With ChatGPT in Medical Education: Qualitative Study

**DOI:** 10.2196/63400

**Published:** 2025-02-20

**Authors:** Noura Abouammoh, Khalid Alhasan, Fadi Aljamaan, Rupesh Raina, Khalid H Malki, Ibraheem Altamimi, Ruaim Muaygil, Hayfaa Wahabi, Amr Jamal, Ali Alhaboob, Rasha Assad Assiri, Jaffar A Al-Tawfiq, Ayman Al-Eyadhy, Mona Soliman, Mohamad-Hani Temsah

**Affiliations:** 1 College of Medicine King Saud University Riyadh Saudi Arabia; 2 Department of Family and Community Medicine King Saud University Medical City King Saud University Riyadh Saudi Arabia; 3 Pediatric Department King Saud University Medical City King Saud University Riyadh Saudi Arabia; 4 Department of Kidney and Pancreas Transplant, Organ Transplant Center of Excellence King Faisal Specialist Hospital & Research Centre Riyadh Saudi Arabia; 5 Critical Care Department King Saud University Medical City King Saud University Riyadh Saudi Arabia; 6 Department of Nephrology Cleveland Clinic Akron General and Akron Children Hospital Akron, OH United States; 7 Research Chair of Voice, Swallowing, and Communication Disorders, Department of Otolaryngology College of Medicine King Saud University Riyadh Saudi Arabia; 8 Medical Education Department King Saud University Medical City King Saud University Riyadh Saudi Arabia; 9 Evidence-Based Health Care & Knowledge Translation Research Chair, Family & Community Medicine Department College of Medicine King Saud University Riyadh Saudi Arabia; 10 Department of Basic Medical Sciences College of Medicine Princess Nourah bint Abdulrahman University Riyadh Saudi Arabia; 11 Specialty Internal Medicine and Quality Department Johns Hopkins Aramco Healthcare Dhahran Saudi Arabia; 12 Infectious Disease Division Department of Medicine Indiana University School of Medicine Indianapolis, IN United States; 13 Infectious Disease Division Department of Medicine Johns Hopkins University School of Medicine Baltimore, MD United States

**Keywords:** ChatGPT, medical education, Saudi Arabia, perceptions, knowledge, medical students, faculty, chatbot, qualitative study, artificial intelligence, AI, AI-based tools, universities, thematic analysis, learning, satisfaction

## Abstract

**Background:**

With the rapid development of artificial intelligence technologies, there is a growing interest in the potential use of artificial intelligence–based tools like ChatGPT in medical education. However, there is limited research on the initial perceptions and experiences of faculty and students with ChatGPT, particularly in Saudi Arabia.

**Objective:**

This study aimed to explore the earliest knowledge, perceived benefits, concerns, and limitations of using ChatGPT in medical education among faculty and students at a leading Saudi Arabian university.

**Methods:**

A qualitative exploratory study was conducted in April 2023, involving focused meetings with medical faculty and students with varying levels of ChatGPT experience. A thematic analysis was used to identify key themes and subthemes emerging from the discussions.

**Results:**

Participants demonstrated good knowledge of ChatGPT and its functions. The main themes were perceptions of ChatGPT use, potential benefits, and concerns about ChatGPT in research and medical education. The perceived benefits included collecting and summarizing information and saving time and effort. However, concerns and limitations centered around the potential lack of critical thinking in the information provided, the ambiguity of references, limitations of access, trust in the output of ChatGPT, and ethical concerns.

**Conclusions:**

This study provides valuable insights into the perceptions and experiences of medical faculty and students regarding the use of newly introduced large language models like ChatGPT in medical education. While the benefits of ChatGPT were recognized, participants also expressed concerns and limitations requiring further studies for effective integration into medical education, exploring the impact of ChatGPT on learning outcomes, student and faculty satisfaction, and the development of critical thinking skills.

## Introduction

Artificial intelligence (AI) is a computer-based technology invented as a digital system to imitate and aid human intellect and skills. The wide use of AI technology is changing the medical field considerably, aiming for more efficient patient management. Medical education is one of the vital domains of health care practice, in which AI has a promising contribution by providing an alternative and efficient means of information access, achieving teaching goals and skills development. As an example, the integration of AI in simulated surgical skills learning showed comparable results compared to remote expert instructions [1], but it led to unintended outcomes in another study, which affected trainees’ efficiency metrics on the cost of safer skills development [2]. Case-based learning is another potential field harnessing AI technology in medical education, which has shown promising results [3]. AI technology has also been used in teaching clinical examination skills, such as breast self-examination, yielding mixed results: high levels of student satisfaction paired with increased anxiety [4]. Such AI-driven interventions will be leading health care practice in the future, such as the introduction of machine-based surgical treatment with robotic surgery, which has effectively promoted diagnostic accuracy, achieving treatment goals and saving health care professionals’ workload [5-7]. AI technology integration in medical education and medical research will not only contribute to patients’ care but also improve if not revolutionize the medical education system [8,9]. All these changes of AI integration into the medical practice need to be accompanied by evolution in the medical teaching and training curricula [8,9], facing significant interest among educators and researchers recently on AI’s rapid involvement in medical education [10-13].

One of the pioneer and popular generative AI-based tools is ChatGPT, a language model developed by OpenAI that uses natural language processing to generate humanlike responses to queries, with many potential applications in health care [14,15]. ChatGPT was perceived by health care workers to positively impact the future of health care systems by 76.7% in a recent study [16]. However, little is known specifically about the perceptions and experiences of faculty and students or trainees against the use of ChatGPT in the context of medical education within Saudi Arabia.

The health care sector in Saudi Arabia is experiencing dramatic growth and reformatting, with a strong emphasis on prioritizing medical education and digitizing the health systems. Therefore, using AI technology in the health care system is a promising strategy for substantial investments in medical, nursing, and other specialized educational disciplines [17]. As medical education evolves, the use of AI-based tools like ChatGPT could potentially transform the way medical education is delivered [18]. Literature has a gap in assessing the perceptions and attitudes of medical education stakeholders regarding integrating AI technology in curricula, clinical teaching, and simulation skills development. Most literature addressed specific AI technology adoption in medical practice or certain educational domains but did not assess it collectively in multiple domains related to medical education. Therefore, it is crucial to explore the medical faculty staff and students’ knowledge, perceived benefits, concerns, and limitations of ChatGPT application in medical education.

This qualitative study seeks to explore the perception on the use of newly introduced AI chatbots, like ChatGPT3.5, in medical education from the perspective of faculty and medical students. By deepening our understanding of faculty and students’ knowledge about ChatGPT and its applications in medical education, this study identifies both the facilitators and barriers to its use. The research offers valuable preliminary insights into the acceptance of AI-based tools in medical education and informs the development of effective strategies for integrating such tools within medical education systems in Saudi Arabia and similar contexts, as more AI models evolve.

## Methods

### Study Design

This study was conducted using a focus group technique at the College of Medicine, King Saud University, a leading university in Saudi Arabia [19]. The study included faculty and students from different levels.

The study aims to preliminarily explore and understand participants’ perceptions of ChatGPT, a newly introduced large language model. A qualitative methodology was chosen, as it is well suited to exploring experiences, meanings, and perspectives from participants’ viewpoints [20-22]. Examining the perceptions of both faculty and students enables a comparative head-to-head analysis of their viewpoints. Qualitative methodology provides a deep explanation of different viewpoints participants may have about ChatGPT use in medical education. It can also allow the authors to propose probing questions to understand and explore users’ perceptions. Although individual interviews would elicit a more detailed picture of an issue, focus group discussion was used, as the aim of the study is to explore different viewpoints using participants’ dynamics and thought sharing to enrich the discussion [23]. Data source triangulation was applied to support the trustworthiness of the findings and allow prelude comparison.

Participants were recruited from the College of Medicine through purposive sampling. As a small number of faculty and students used ChatGPT at the time of data collection, a purposive sample was applied. A student was asked to announce the need to interview students who have ever used ChatGPT. Another announcement to faculty was made, and an invitation was sent to random faculties from 3 departments who use or want to share their ideas about ChatGPT in medical education. The sample included 6 medical faculty members (2 associate professors and 4 professors) and 6 medical students (2 second year, 2 third year, 1 fourth year, and 1 fifth year). Two focus group discussions were conducted in April 2023 on the Zoom platform (Zoom Video Communications), one with faculty members and the other with students, and each group consisted of 6 participants. The discussions were conducted in English language as preferred by the participants. Two of the authors (NA and MHT) served as moderators, and each discussion lasted for approximately 1 hour.

Using the Zoom platform in data collection facilitated gathering participants at the same time after working hours. As the team acknowledged that nonverbal cues may not be detected as participants refrained from opening their cameras, follow-up questions and probing were used to minimize subjectivity in understanding participants’ responses. Not all participants knew each other; hence, the setting was more private to freely share opposing views.

A topic guide was prepared by the author (NA) to cover aspects such as participants’ familiarity with ChatGPT, its uses, facilitators, and limiting factors of its incorporation in medical education. Probing and follow-up questions were allowed depending on participants’ responses. The themes were saturated at that time after the second interview possibly due to the limited experience of participants in the early stages of ChatGPT launch. Thematic analysis was used to analyze the data using a priori themes and allowing new themes to emerge from the data [24]. The discussions were transcribed using Zoom’s automatic transcription feature. This feature had the advantage of identifying the name that the participants chose for themselves in the discussion and linking it with the speaker.

The transcripts were revised and read multiple times to identify patterns and themes that emerged from the data. A coding framework was developed from the data by each coder (NA and MHT) and applied using NVivo software (version 12; QSR International) [25]. Themes were identified and refined through an iterative process of coding, reviewing, and discussing the data among the research team until a consensus was reached [24]. Initial codes were developed by 2 different authors (NA and MHT) and then, comparison and discussion were made to agree on the coding framework. Coding themes were similar, and no major changes were made in the thematic framework.

### Ethical Considerations

This study received ethics approval from the Institutional Review Board at King Saud University (approval 23/0155/IRB). All participants provided verbal informed consent prior to their inclusion in the study, including consent for the audio recording of interviews. Participants were fully informed about the purpose of the study, the voluntary nature of their participation, and their right to withdraw at any time without any consequences. To ensure participant privacy and confidentiality, pseudonyms were assigned, and no identifying information was included in the transcripts or final report. The data were securely stored and accessible only to authorized members of the research team. No compensation was provided to participants for their participation in the study.

## Results

### Overview

**Figure 1:**
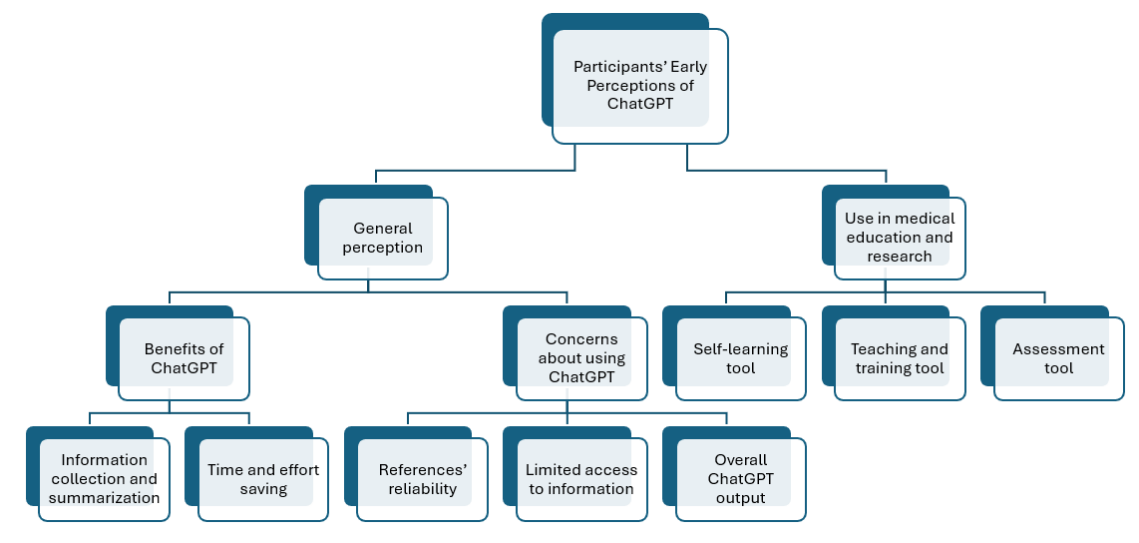
Thematic framework of participants’ perception on using ChatGPT.

In total, 6 medical faculty staff and 6 medical students with different experiences with ChatGPT participated in the study. Table 1 shows their demographic data. Figure 1 displays the thematic framework used to assess participants’ perception of ChatGPT in general and in medical education.

Analysis of the data from the discussion generated two main themes: (1) participants’ general perception of ChatGPT and (2) ChatGPT use in medical education and research.

**Table 1:** Participants position, department, and frequency of ChatGPT use.

Participant code	Position	Department	Age (years) and sex	Using ChatGPT
Participant 1	Faculty	Critical Care Department, College of Medicine	44 and male	Regular user in medical education
Participant 2	Faculty	Ear, Nose, and Throat department, College of Medicine	56 and male	Regular user in medical education
Participant 3	Faculty	Family medicine, College of Medicine	60 and female	Not a user
Participant 4	Faculty	Pediatrics department, College of Medicine	38 and male	Not a user
Participant 5	Faculty	Pediatrics department, College of Medicine	41 and male	Regular user in medical education
Participant 6	Faculty	Medical Education Department, College of Medicine	58 and female	Not a user
Student 1	Student	College of Medicine	20 and male	Regular user for general search
Student 2	Student	College of Medicine	22 and male	Regular user for general search
Student 3	Student	College of Medicine	21 and male	Regular user for general search
Student 4	Student	College of Medicine	21 and male	Regular user for general search
Student 5	Student	College of Medicine	19 and male	Regular user for general search
Student 6	Student	College of Medicine	20 and male	Regular user for general search

### Participants’ General Perception of ChatGPT

#### Overview

All participants expressed good knowledge of ChatGPT’s main goal and functions. One participant noted:

The idea from this software is that it will chat with you regarding any topic you will ask about...it chats with me in a human like manner, and collect for me the answers from all over resources, and display them.

Participant 1

One student described ChatGPT as an “assistant,” and others elaborated:

Artificial intelligence helps me execute the command that I’m asked to execute.

Student 2

It’s another way of searching for highly accurate information, depending on what I search for and how I search for it.

Student 5

Participants were challenged about ChatGPT compared to other traditional search engines: “It is not at similar to Google, even Google started invention of AI application to enrich its platform” (Participant 2). Most participants supported the use of ChatGPT but were not concerned about its information sources.

#### Benefits of ChatGPT

Two main subthemes emerged from the focus group discussions about the benefits of using ChatGPT.

#### Collects and Summarizes Information

The majority of the participants believed that searching for information through ChatGPT is more efficient compared to standard search engines, as the former saves time by summarizing and textualizing the raw information output from the search: “ChatGPT is beautiful in collecting information and presenting it to me in a simplified text that I can easily comprehend” (Participant 2).

Few participants used ChatGPT to review scientific papers or provide ideas for new papers: “I used it to study limitations of studies and the future recommendations for studies I was asked to review...it gives me ideas” (Participant 2).

Students also noted:

I find ChatGPT more directive towards what I ask, and to the point, mostly because when I look for something on classical search engine, such as Google...I have to go into some sub web pages which has an answer and look between all the thousands of answers to find one. While ChatGPT will give it to me concisely like this is option A, option B, option C.

Student 3

Another faculty added, “It will do the search for me; then even with critically appraise it and give me the final result” (Participant 1). However, one faculty participant was more conservative in her comments about using AI in collecting data and did not perceive the information displayed by ChatGPT as reliable because it lacks the “critical thinking” skill to enable it to reach a final scientific plausible conclusion, “The problem of collecting all the information in one place is that collecting the information and giving it in a nutshell, in one place. This machine is not critically thinking” (Participant 3).

#### Saves Time and Efforts

Opinions varied in terms of whether using ChatGPT saves time and effort, considering the perceived benefits. One faculty mentioned: “It saves time when I’m stuck in generating exam question” (Participant 2). A student added: “It’s not accurate, but at least it saves me time. This is the most important point” (Student 4).

On the other hand, another faculty participant subtly disclosed her denunciation about the functionality of ChatGPT. She believed that ChatGPT helps partly in performing tasks, but that advantage is contradicted by paying time to verifying and authenticating the ChatGPT output.

Me as a researcher. When I search for information I’m putting it together, ChatGPT tries to put it for me. So far, I can’t see it superior to the human mind...It does some of the work for me, but I have to take it with a bunch of salt.

Participant 3

#### Concerns About Using ChatGPT

When the participants discussed the drawbacks of using ChatGPT, they mentioned expressions such as “hallucination” and “blinding euphorically.” The following subthemes emerged as perceived drawbacks of ChatGPT.

#### References Reliability

While few participants were not sure about the source of ChatGPT information, most participants believed that it is the internet: “It’s the same data retriever as Google” (Participant 3). Other participants had a deeper view: “ChatGPT generated references, and citations have to be taken with caution” (Participant 2).

Faculty experienced situations where they doubted the reference of the information provided by ChatGPT. For example, one faculty noted: “I’m not sure what are the sources used to extract information, even if I ask for references, it might not mention them...or at least it will not volunteer in mentioning them” (Participant 1). While others defended that: “If it doesn’t have access to the reference, it will tell that it doesn’t have access, but if the reference is online, it can refer to that” (Participant 2).

Similarly, a student commented: “It is multitasking, rather than searching for the source of information, it presents the answer and references” (Student 5). One participant pointed that the unreliability of ChatGPT sources supports her view of not relying on ChatGPT.

#### Limited Access to Information

Several participants acknowledged ChatGPT’s limitation in accessing all available information, driving caution while using ChatGPT:

One of the restrictions regarding medical search, it’s restricted to certain resources like PubMed...there are some other medical websites that it cannot access yet.

Participant 4

We don’t know the algorithm behind the search nor exactly how it looks for information.

Participant 6

One of the participants elaborated that ChatGPT is invented by humans; therefore, they may manipulate or restrict its search and output.

It’s not free of bias. If I am asking for something morally wrong or illegal. It will not answer because it is constrained. So, it is not fully free from human constraints.

Student 3

Some faculty participants raised an ethical concern that may affect the trust in ChatGPT information. One participant explained:

Can drug company pay ChatGPT to display answers that are in favour of certain medication? Could ChatGPT be manipulated? ChatGPT inventors are for sure looking for money somehow by anyway!

Participant 1

#### Overall ChatGPT Output

All participants believed that ChatGPT users should not fully trust the information presented and practice caution, while others elaborated that it is ideal for new topics as a jumpstart:

I should not take it (information from ChatGPT) for granted; I have to review what’s there, but it gives me a nice idea, very excellent ideas...It sheds the light on some certain angles that I was not looking for.

Participant 2

Some participants pointed that trusting ChatGPT output depends on your previous background about the topic:

I should have the ability to differentiate between what is reliable and what is not reliable...Myself, I am not well-versed in medical education. For example, I am highly qualified in research, but regarding education I take for granted whatever output from ChatGPT in that regard, while I can filter information regarding research and judge it well.

Participant 3

One student agreed:

It depends on what I am looking for. Sometimes it’s very accurate. Sometimes it’s not...But as a human mind I have an idea about what I am looking for, therefore, I can judge if its accurate or doubt the answer.

Student 4

All participants agreed that the unfamiliarity of ChatGPT users with its search algorithm enforced the participants’ trust issue.

A faculty explained:

Do we know the ChatGPT searching methodology? is it scientific methodology? How it extracts the information from the paper, how it appraises it? What are the sources that this engine has access to? All this will augment the reliability of my experience.

Participant 1

One participant mentioned that ChatGPT cannot be used for critical thinking in certain contexts; thus, it cannot be fully trusted:

It cannot give me what is relevant to me, my community and population and my students...It might be dangerous to put ChatGPT superior to human intellect!

Participant 3

Another faculty participant defended the ChatGPT’s reliability, noting that it declares its level of expertise and specialty ahead of each information presented:

If I ask ChatGPT about something in geology, it will start with “I am not a geologist” and then move on with the dialogue...and it finishes the response by “it is very important to refer to those sources.”

Participant 2

### ChatGPT Use in Medical Education and Research

Participants discussed ChatGPT use in medical education from 3 aspects as discussed below, but in general, they raised concerns about using it without appropriate and dedicated training.

#### Self-Learning Tool

The majority of faculty participants supported using ChatGPT in the teaching process. A faculty participant commented:

Students are no longer enjoying the usual long lectures, or didactic lectures but they enjoy more challenging aspects exploring a new experience, and living it...I think the ChatGPT could be used as a very good trigger for the students to go and read and find out more, discuss among themselves and go explore this with their seniors, with their educators.

Participant 5

Another faculty added that it should be used to get an idea about a topic, but further reading is important for students:

ChatGPT is like a short fast access to a topic, it helps to get the most important information...they (students) need to read the references.

Participant 1

However, another faculty participant raised concerns using ChatGPT for concluding opinions and summing debates:

If they (students) use ChatGPT just for recalling information then no problem...But if they want to make inferences, they should not use it.

Participant 3

ChatGPT methodology was raised by another faculty participant who did not support using it in learning at all because of its unclear methodology and unverified information sources.

On the other side, the majority of student participants did not support using ChatGPT to obtain information and felt the traditional search engines are more reliable and easier to use:

I do not perceive it as a search engine. I don’t look up medical information on it, or anything, because I find the classic search engines easier.

Student 2

I know exactly where the reliable sources are. Then I can take the information from other sources with confidence, and more simple steps.

Student 1

Some participants, while supportive of ChatGPT’s use in medical practice, emphasized its role in clinical medicine education. They raised concerns about its impact on decision-making, particularly due to ChatGPT’s inadequate or unclear strategy for disclosing information sources:

If I look at the other search engines for which support medical information, they present like up-to-date information...ChatGPT is very complex, and the methodology and the algorithm it uses is not clear so, it is not a reliable source of information for decision making and for serious information.

Participant 1

The issue of updated sources in ChatGPT was also raised:

We need to be cautious about using the information...the medical field information is changing very quickly, so we have to be careful about this point.

Participant 4

Other participants debated that the information accuracy depends on user searching and prompt engineering skills:

Prompt questions will make the difference in getting the response, and I recommend digging into the prompts technology to get more accurate answers, and doing this is important to acquire the right answer.

Participant 2

You get the response according to the precision of the search.

Student 1

Interestingly, a faculty participant raised the concern of students and faculty losing their critical thinking skills if they depend on ChatGPT:

It is dangerous...because we are replacing critical thinking. We are prioritizing this thing over human intellect.

Participant 3

A student participant who expressed poor research skills was concerned about such skills being affected or even weakened by dependence on ChatGPT in research. In general, students did not support the use of ChatGPT as the primary source of information, especially for new topics, but as a collateral resource.

#### Teaching and Training Tool

Some participants believed that teaching modalities should change after the introduction of AI technology. They expressed optimism of more teaching methodology shifting from memorization to critical thinking; however, this aim was not perceived achievable through ChatGPT so far:

We must invest more in the skills of our medical students and problem-solving critical thinking analysis. These are the areas that is lacking in the ChatGPT, and that we need to focus more on.

Participant 4

Faculty participants raised concerns about students’ replacement of traditional lectures with AI applications like ChatGPT, which might be risky in general, especially in the current stage of unverified and undedicated AI applications for medical education.

Another concern raised from one faculty regarding the lecturers and trainers:

Do our faculty have enough knowledge to use and recommend ChatGPT for their students and instruct them how to use it and get maximum benefit from it?

Participant 3

However, all students did not see themselves relying on ChatGPT for learning: “We just need to be familiar on how to use ChatGPT and use it as a tool that supports our search rather than completely relying on it” (Student 2)*.*

Faculty participants differentiated between the needs of postgraduate and undergraduate students and their use of ChatGPT. One faculty (Participant 3) felt that using AI in training postgraduate trainees would be difficult because postgraduate training depends on building skills, while undergraduate depends on memorization as per him.

ChatGPT might be a tool to generate clinical scenarios and draw a framework for discussions with the students:

One problem would take weeks from our team and long hours of sitting together and creating the medical problems that we teach in the problem-based learning sessions. So, it would be interesting to see how ChatGPT deals with this.

Participant 4

An interesting point mentioned by some faculty is the inability of ChatGPT to teach students human, emotional, and social skills: “Using AI is not designed to help in teaching some skills such as Humanity and the communication, the teamwork” (Participant 4).

#### Assessment Tool

Most faculty participants mentioned using ChatGPT for academic assessment like examination questions generation:

I asked ChatGPT to generate questions for me with scenario and without scenario...it was good to Very good. It’s not reaching to excellent level. I have to review and modify.

Participant 2

In addition, most faculty participants mentioned using ChatGPT for medical problems, clinical scenarios, and bedside teaching. Some faculty participants raised the idea of using AI applications like ChatGPT to assess the quality and objectives of examinations in order to guide certain questions to assess critical thinking rather than recall knowledge only. Cheating and plagiarism were one of the raised concerns by the faculty during the discussion: “We have to be very careful about cheating and misuse of ChatGPT by our medical students in medical assignments” (Participant 4).

In line with the former comment, one student defended his use of ChatGPT, raising a debatable point of using AI applications for academic assignments is ethical or not:

I mainly use it for writing, and then I just review it and edit it...mainly for research or some essays...for example I’d give it some data, and I ask it to write a paragraph that summarizes this data, or an introduction to something for example (Disease X).

Student 2

Another student mentioned that his use of ChatGPT in assignments is mainly for summarization. Others use it to collect information resources: “It can make my job way easier. For example, if I have a research assignment to just collect the resources about a topic” (Student 3).

Overall, all participants reached a conclusion of being open-minded and accepting for the ChatGPT intrusion into our lives: “I think it’s coming in the near future, and we need to live in the reality to adjust and take the best out of it” (Participant 4).

## Discussion

### Principal Findings

This paper presents a general snapshot of the faculty and undergraduate medical students’ perceptions of ChatGPT and its use in medical education. All participants demonstrated a good understanding of ChatGPT and its functionalities; some described its role as assistive, while others found it as a mere information search tool. Almost all participants were impressed by ChatGPT’s ability to provide a concise summary of search results compared to traditional search engines, which is in line with the literature [26]. On the other hand, few students in our study perceived Google as a better tool for learning.

In line with other publications [27], our participants believe that ChatGPT provides a more user-appealing and faster solution for busy users by delivering a summarized, high-caliber textual output. One of the major challenges they mentioned regarding ChatGPT use is its sources of information, which is in line with previously published similar studies showing that students and faculty are aware of the limitations of ChatGPT that influence its accuracy [26,27]. In a comparative study between platforms, ChatGPT-generated responses were considered to be reliable and beneficial, while others deemed them potentially risky [28]. For example, a study showed that there were concerns about ChatGPT advice regarding antimicrobial stewardship, general course lengths were accurate but the duration varied, and source control was either incorrectly cited as justification for prolonging therapy or ignored entirely [29]. Therefore, ChatGPT output should be dealt with skeptically and selectively, as poor users’ baseline knowledge might lead to risky, dangerous, or suboptimal conclusions. Previous literature has shown that in comparison with Google, the majority of the participants tend to doubtfully trust ChatGPT output for reasons related to the novelty of AI and users, lack of understanding of its algorithm, and information sources as studied previously [14,30]. Notably, only 40% of these experts concluded that the perceived value of ChatGPT’s responses outperformed those from Google [31].

Therefore, participants tend to trust ChatGPT responses if they have a previous background about the search topic. Participants suggested that while ChatGPT might be helpful in certain aspects of medical education, users should approach the information with caution and apply their medical judgment.

The participants’ concerns about ChatGPT output also stemmed partly from the observed phenomenon of references’ hallucinations, which raised serious concerns about its reliability and validity [32-34]. In addition, they stressed on the point of ChatGPT’s limited access to updated medical literature. A previous study had cautioned authors regarding references generated by ChatGPT [35]. To overcome these limitations, developers should work on expanding the access of ChatGPT’s resources, improving its search methodology, and ensuring a more comprehensive and reliable source of information.

Faculty participants explored the potential of ChatGPT in generating examination questions and clinical scenarios, enhancing bedside teaching, and reviewing assessments. Still, they emphasized the need for reviewing and modifying AI-generated content as well as the importance of developing policies and strategies to tackle potential academic misconduct related to ChatGPT use. Previous studies showed ChatGPT’s excellent performance as it passed the American Heart Association examination with 84% accuracy, but it failed Taiwan’s family medicine examination and fared poorly on the urology self-assessment examination [36-38]. A study concluded that ChatGPT responses were frequently incomplete and sometimes misleading [26]. However, a recent expletory review showed that ChatGPT has a potential impact on medical education, scientific research, and medical writing [14]. Thus, the ChatGPT’s generated questions need to be carefully examined and revised especially regarding scientific content. Other research highlighted that generated output in that regard is not highly different among different AI platforms, as the multiple-choice question–based examination performance of ChatGPT was marginally better than that of Google’s Bard [39].

Both faculty and students appreciated the time-saving advantage of ChatGPT and its fast access to information. Therefore, faculty used it in preparing lecture materials and examination questions. While students used it in their academic assignments, this mirrors a previous study about ChatGPT perception among students who used it for generating academic content, brainstorming ideas, and writing texts [40,41].

Faculty in our study and previous research raised concerns about students’ ChatGPT overuse [13,27,42]. According to our participants, using it by students may interfere with their critical thinking, writing, and information retrieval skills. Faculty highlighted the students’ need to critically review and modify the AI-generated content, ensuring it aligns with academic standards and expectations. Banerjee et al [11] reported that postgraduate trainee doctors have an overall positive perception of the impact of AI on clinical training; however, they found that AI will eventually reduce the trainees’ clinical judgment and practical skills. In line with that, the faculty participants were concerned about students’ self-reliance on AI applications on the cost of traditional teaching methods, which might deprive them from skills best learned in person or group teaching. One study listed the following as disadvantages: lack of originality, inaccurate content, or unknown data sources [14]. It is also uncertain how ChatGPT handles offensive material, false information, or plagiarism [34].

Ethical concerns, such as potential manipulation by pharmaceutical companies, were raised by participants. Maintaining transparency and integrity in AI-generated information is vital to address these concerns. Implementing measures such as third-party audits, strict guidelines, data transparency, and continuous monitoring of ChatGPT’s information sources can help ensure the unmanipulated ethical use of ChatGPT in medical education [43-45].

We recommend creating guidelines for students on the appropriate use of AI applications, specifying tasks they should complete independently and the extent to which AI tools can assist. Additionally, we propose incorporating teaching sessions to help students critically evaluate AI-generated outputs. At this early stage of AI adoption [46], group teaching sessions comparing the critical appraisal of medical topics using AI tools versus traditional search methods would be beneficial. We also emphasize leveraging AI applications primarily as advanced search engines and using their summarization capabilities rather than relying entirely on their final outputs.

Participants emphasized the importance of being open-minded and adopting new technologies like AI chatbots including ChatGPT. As AI chatbots could have cultural bias, addressing cultural differences in learning styles is vital [46,47].

The potential implications of using ChatGPT in medical education include improved efficiency, streamlined information gathering, and time-saving benefits. However, future research is needed to explore the impact of AI-based tools on medical education in terms of quality, student and faculty satisfaction, and the development of critical thinking skills. Ongoing research and evaluation are essential to ensure the effective integration of AI-based tools like ChatGPT into medical education while addressing potential concerns and limitations.

In preparation for the future of medical education, educational institutions should be proactive in integrating AI technologies like ChatGPT into their curricula and teaching methodologies [48,49]. Educators and policy makers need to remain vigilant about reliability concerns and actively take steps to be ready to address the ethical challenges and possibilities arising from the use of AI in health professions education [37,45]. This process should involve regular evaluations, ongoing improvements, and a strong emphasis on maintaining the essential human aspects of medical education, such as critical thinking, communication, and empathy.

### Strengths

One of the strengths of this study is the qualitative design, which allowed for an in-depth exploration of participants’ experiences, perceptions, and concerns related to the use of ChatGPT in medical education, revealing diverse viewpoints and generating valuable insights into the potential benefits and challenges of integrating ChatGPT into medical education [50]. Moreover, the study involved participants with varying levels of experience with ChatGPT, ensuring a comprehensive understanding of the perspectives of both novices and experienced users. The identification of themes and subthemes has laid a solid foundation for further research and exploration of AI-based tools like ChatGPT in medical education.

### Limitations

There are some limitations to our study. The sample size was relatively small, and the participants were primarily drawn from a single institution, which may limit the generalizability of some findings to other medical education settings. The study did not quantitatively assess the impact of ChatGPT on learning outcomes, satisfaction, or other measurable aspects of medical education, which could in the future provide valuable data to supplement the qualitative findings. Additionally, since the study’s focus was on understanding the perception of faculty and students, the perspectives of other stakeholders, such as administrators and policy makers, were not captured, and this could be explored in future research [51-53]. Furthermore, the study, which was conducted in the early phase of ChatGPT launching, did not explore the long-term implications and potential changes in perception and use of ChatGPT over time, as participants’ experience with the tool may evolve, altering their views on its benefits and limitations [54].

Therefore, future research should incorporate larger and more diverse samples from multiple institutions as well as conduct quantitative studies to measure the impact of ChatGPT on various aspects of medical education in Saudi Arabia specifically and globally. Longitudinal studies could be conducted to assess the changes in perception and use of ChatGPT over time and evaluate the long-term effects of its integration into medical education.

### Conclusions

Participants praised the advantages of ChatGPT, such as time-saving and excellent summarizing skills. However, concerns were raised regarding the accuracy and critical appraisal of information provided by ChatGPT and the need to approach the information with caution. ChatGPT-delivered information and cited references’ hallucination were concerns seriously raised by participants, which needs urgent assessment and solution in addition to limited access to certain medical databases. This study highlights the need for ongoing research and evaluation to ensure that AI-based tools like ChatGPT are effectively integrated into medical education while addressing potential concerns and limitations. Educators and students must also maintain a strong foundation in critical thinking and judgment. As medical education continues to evolve, the integration of AI technologies like ChatGPT has the potential to transform the way medical education is delivered but must be done with a thoughtful and ethical approach.

## Abbreviations

AI: artificial intelligence

## Data Availability

The preprint of this research is available [56]. The datasets generated and analyzed during this study are not publicly available due to institutional review board privacy regulations but are available from the corresponding author on reasonable request after obtaining institutional review board approval.
